# The Impact of Positivity and Parochial Altruism on Protective Behaviours during the First COVID-19 Lockdown in Italy

**DOI:** 10.3390/ijerph191610153

**Published:** 2022-08-16

**Authors:** Claudio Singh Solorzano, Maria Serena Panasiti, Alessandra Di Pucchio, Caterina Grano

**Affiliations:** 1 Department of Psychology, Sapienza University, 00185 Rome, Italy; 2 IRCSS, Santa Lucia Foundation, 00142 Rome, Italy; 3 Training Office, Istituto Superiore di Sanità, 00161 Rome, Italy

**Keywords:** COVID-19, positivity, parochial altruism, protective behaviours, social distancing behaviours, hygiene behaviours

## Abstract

Implementation of COVID-19 protective behaviours, such as social distancing or frequent hand washing during the lockdown, was critical to prevent the spread of the COVID-19 pandemic. In this cross-sectional study, we examined the effect of positivity and parochial altruism on implementing COVID-19 health-protective behaviours during the Italian lockdown. A sample of 460 participants completed an online questionnaire that included demographic measures, Positivity Scale and COVID-19 measures of health-protective behaviours. To measure parochial altruism, we used a hypothetical dictator game played with others who could vary in their social distance from the participants. Results showed that participants in the hypothetical game gave more money to parents and siblings than to best friends, cousins, neighbours, and strangers. Furthermore, both positivity and parochial altruism (more altruism toward close vs. distant people) were positively associated with implementing hygiene behaviours but not with social distancing. Finally, mediation analysis showed that increases in parochial altruism mediated the effect of positivity on hygiene behaviour. These findings extend knowledge about the factors beyond the implementation of COVID-19 health-protective behaviours during a lockdown situation.

## 1. Introduction

Globally, more than 536 million people contracted coronavirus disease (COVID-19), and over 6.3 million deaths were reported at the time of writing [1]. Since 9 March 2020, a total of 18,213 million COVID-19 cases have been reported in Italy, including an estimated 165,000 deaths [2]. To respond to this situation, the Italian government issued some decrees to avoid the spread of COVID-19, including the country’s lockdown (from 10 March until 4 May) [3,4,5]. This decision led to the closure of educational institutes and shops selling non-essential products, travel restrictions, stay-at-home orders (i.e., individuals had permission to leave their homes only for demonstrated necessities, such as for health reasons, shopping for basic needs, and work), and social distancing and hygiene instructions were recommended [6,7]. The implementation of COVID-19 protective behaviours such as social distancing or frequent handwashing during the lockdown was crucial in preventing the spread of the virus [8]. Recent studies indicated that social cognitive constructs such as attitudes, perceived efficacy of the proposed measures, and self-efficacy influenced the implementation of preventive measures [9,10,11]. Negative factors have also been reported to be related to the engagement of COVID-19 preventive behaviours, such as anxiety, depressive symptoms, and fear of the virus [7,12,13]. Less is known about how positive orientations (such as positivity or altruism) can influence respect for recommended protective measures.

*Positivity* (or *positivity orientation*) is a general tendency to view, interpret, and positively evaluate various life domains, including oneself, one’s future, and past experiences [14,15,16]. Previous studies showed that positivity is the common latent dimension underlying three more specific constructs: self-esteem, life satisfaction, and dispositional optimism. The construct of positivity has higher predictive power and accounted for more variance than the separate specific constructs [17,18]. Moreover, twin studies indicated that self-esteem, life satisfaction, and dispositional optimism shared a large variance due to genetic factors (between 0.80 and 0.87), suggesting that positivity might be a common genetic factor affecting these three constructs [19,20]. According to current evidence, positivity is a basic disposition needed to effectively deal with the difficulties and challenges of human conditions [14]. For instance, higher positivity was associated with higher levels of adaptive and well-functioning characteristics, such us ego-resilience, self-efficacy, positive interpersonal relationships, prosocial behaviours, adaptive coping styles, and an active concentration on the present [14,18,21]. Furthermore, greater positive orientation was related to less use of tobacco and alcohol in adolescence [22], fewer feelings of exhaustion and problems with physical mobility in older adults [23], and fewer psychological and physical dysfunction symptoms in cancer patients [24]. These findings suggest that positivity may play a role in the optimal functioning of persons by equipping them with coping responses which favour more adaptive reactions towards adversities. Recently published studies reported that positivity mediated the link between COVID-19-related perceived stress and psychological health (i.e., death distress and happiness) [25]. However, its relationship with COVID-19 health-protective behaviours is still unexplored.

*Prosociality*, the tendency to behave in a way that is intended to benefit others, may also affect the propension to engage in COVID-19 protective behaviours (e.g., hand washing, social distancing, wearing face masks). A recent review of the psychological impact of the quarantines during different outbreaks indicated a strong association between altruistic motivations, better mental health outcomes, and compliance with quarantine advice [26]. A high motivation to comply with pandemic indications regarding preventive behaviours, such as mask-wearing [27], could not only depend on selfish goals (i.e., protection for oneself) but also on altruistic ones such as reciprocity concerns [28,29]. One recent study reported that a prosocial emotional process (i.e., empathy for people who are vulnerable to the virus) increased the likelihood of wearing a face mask and maintaining the proper physical distance from others [30]. Moreover, Campos-Mercade and colleagues [31] showed that the more people avoid putting others at risk for their personal benefit, the more they are likely to follow COVID-19 protective behaviours (e.g., following physical distancing, staying home if sick, buying face masks).

Interestingly, prosocial behaviours tend to decrease when the social distance between donor and recipient (i.e., social discounting) is higher and increase with members of one’s social group (i.e., parochial altruism) [32,33,34]. This pattern has been documented even during the COVID-19 lockdown, with adolescents donating more money to familiar than unfamiliar peers but showing the highest level of altruism to deserving targets such as doctors [35]. However, whether the implementation of protective behaviour during the lockdown was related to the tendency to be more altruistic towards the close (e.g., family members) or distant ones (e.g., strangers) was still unclear.

This study aimed to explore the effect of positivity and parochial altruism on implementing COVID-19 health-protective behaviours during the Italian lockdown. We reasoned that since positivity exerts a crucial adaptive function by promoting more effective coping strategies, better social relationships, and higher positive emotions, it could be strongly associated with adherence to preventive behaviour. Regarding the relationship between parochial altruism and protective behaviours, we had a more exploratory approach since we believed that, on the one hand, preventive behaviours could have been motivated by the intention to protect close ones; on the other hand, altruism toward distant others (and thus society in general) could also be a potential trigger for such behaviour. Finally, we tested whether having a higher positivity (and thus a better way to cope with adversity) could have a role in modulating parochial altruism (by enhancing or reducing altruism towards close ones) and whether that, in turn, could have an impact on the engagement in COVID-19 protective behaviours.

## 2. Materials and Methods

### 2.1. Study Design and Participants

A cross-sectional web-based survey design was adopted. Participants were recruited from the general population during the COVID-19 quarantine in Italy. Data collection was administered through a Qualtrics online questionnaire which was enabled from 8 April to 3 May 2020. Potential respondents were invited via e-mail and text messages in which the study was briefly described, and the link to the questionnaire was included. All participants took part voluntarily and were not remunerated. To avoid incomplete or inconsistent questionnaires, the study was not posted on social media. Before starting the survey, participants read more information on the purpose of the study and data confidentiality and were asked to give their informed consent. Respondents were included in the study if they were over 18 and lived in Italy during the lockdown. People not living in Italy at the time of the recruitment were not included. There were no other exclusion criteria. A total of 460 participants provided complete and valid data and were included in the analyses. This study was conducted according to the Declaration of Helsinki. The protocol was reviewed and approved by the Institutional Review Board of the Psychology Department, Sapienza University of Rome (Prot. No. 0000587 of 31 March 2020).

### 2.2. Measures

#### 2.2.1. Demographic Characteristics

Participants’ age, gender, education, marital status, occupation, household size, and self-reported health status were collected. Education was scored as categorical variables with three groups: high school graduates or lower, college graduates or students, and post-graduate or higher. Marital status was defined as a categorical variable with three groups: single, married or living with a partner, and not married (i.e., widowed, divorced, or separated). Occupation was defined as a binary variable for workers (i.e., full, or part-time) and not-worker (i.e., unemployed or pensioner). Household size was measured as the number of people living in the household (including the participant). Self-reported health status was assessed using the first item of the Italian version of the 36-Item Short Form Survey [36]. The item (i.e., “In general, would you say your health is:”) was rated on a Likert-type scale between 0 (poor) and 4 (excellent).

#### 2.2.2. Positivity

Positivity was measured using the 8-item Positivity Scale (P Scale), a scale designed to measure the common latent dimension between self-esteem, life satisfaction, and dispositional optimism [15]. Each item is scored on a 5-point scale ranging from 1—strongly disagree to 5—strongly agree. Sample items are “I generally feel confident in myself”, “I have great faith in the future”, and “I am satisfied with my life”. Responses were summed to produce a total score, ranging from 8 to 40, with higher scores indicating greater positivity orientation. The P Scale had good internal validity and reliability across different languages, cultural contexts, and developmental phases [14], and it was used in other studies during the COVID-19 pandemic [25,37]. The Cronbach’s alpha of the questionnaire in this study was 0.82.

#### 2.2.3. Impact of Social Closeness on Altruism (ISCA)

Altruism was tested through 6 trials of a dictator game in which participants (dictators) were asked to split a hypothetical sum of EUR 100 [38] between themselves and six different recipients: (1) a sibling; (2) a parent; (3) a cousin; (4) a best friend; (5) a neighbour; and (6) a stranger. Dictators had to imagine a hypothetical situation, as realistically as possible, in which they received EUR 100 and had to decide how to split this money between themselves and one of the recipients. They could decide whether to keep all the money for themselves or to share this money with the other person in any way they desired. Dictators had to report the amount of money they wanted to donate to each recipient in a visual analogue scale (VAS) ranging from EUR 0 to 100. The impact of social closeness on altruism (ISCA) index was obtained as follows: the category to which participants donated the most (as perceived as the socially closest) and the least (as perceived as the socially most distant) were identified, and then a subtractive index between these two categories was calculated for each participant. Positive values for this index reflected people who donated more to *close* than *distant* others; values around zero indicated people who donated similar amounts to these two categories; negative values indicated people who donated more to distant than close others. This measure has previously been shown to correlate to real-life altruistic behaviour such as umbilical blood cord donation [39].

#### 2.2.4. COVID-19 Protective-Health Behaviours

Preventive behaviours were assessed through 8 questions based on the Italian Ministry of Health’s recommendations to be followed during the Italian quarantine [40]. The questions have been grouped equally into two different measures: hygiene behaviours (i.e., preventive behaviours) and social distancing behaviours (i.e., avoidance behaviours). Protective hygiene behaviours were evaluated by asking the participants to evaluate the truthfulness of these statements: “In the last week… I wore a mask when I was outside”, “… I washed my hand more often than usual”, “… I used disinfectants to clean the house”, and “…I washed my clothes right after I returned home”. The social distancing protective behaviours were evaluated by asking the participant to evaluate the truthfulness of these statements: “In the last week I avoided travelling by plane, train, taxi and metro”, “… I avoided going in crowded places”, “… I avoided shaking hands with other people”, and “… I avoided going shopping more often than once a week”. Each item was rated on a Likert-type scale between 0 (not at all true) and 4 (completely true). Responses for each category of protective behaviours (i.e., hygiene vs. social distancing) were summed to produce two total scores ranging from 0 to 16. Higher scores mean greater preventive/avoidance behaviours during the COVID-19 pandemic. The Cronbach’s alpha was 0.64 for the protective hygiene behaviours scale and 0.59 for the social distancing protective behaviours scale. The mean inter-item correlation was 0.32 for the protective hygiene behaviours scale and 0.27 for the social distancing protective behaviours scale.

#### 2.2.5. Statistical Analysis

Data analyses were conducted using IBM SPSS Statistics version 24 (SPSS Inc., Armonk, NY, USA). Descriptive statistics are expressed as means ± standard deviations or as the number of participants with the percentage in parenthesis. Cronbach’s alphas were computed for all the used scales. The Cronbach’s alpha value for exploratory studies should be 0.60 or above for moderate reliability [41,42]. However, with a small number of items in the scale (i.e., fewer than 10), low Cronbach’s values such as 0.50 are commonly found [42,43]. To better evaluated the reliability of the COVID-19 protective behaviours scales, we also reported the mean inter-item correlation for the items of each scale. Optimal mean inter-item correlation values range from 0.2 to 0.4 [44,45]. The amount of euros donated to each recipient was analysed through a one-way repeated measure ANOVA with recipient (sibling, parent, cousin, best friend, neighbour, stranger) as a within-subject measure. Pearson’s correlations were run to explore the relationship between the variables of interest. To examine whether the ISCA index and altruistic behaviours would mediate the relationship between positivity and protective behaviours, bootstrap mediation analysis for simple mediation through the SPSS PROCESS macro was applied [46]. For coefficient and indirect estimation, 5000 bootstrap samples were used, and 95% bias-corrected confidence intervals (CI) for the indirect effect were reported in the results. The SPSS PROCESS macro was used instead of structural equation modelling (SEM) to test this mediation model because it is a reliable and widely used technique that provides similar results to SEM by implementing bootstrapping methods [47,48,49]. In the simple mediation model, the independent variable was positivity, and the dependent variable was preventive hygiene behaviours. The mediator of the models was the impact of social closeness on altruism.

## 3. Results

### 3.1. Descriptive Analyses

Descriptive characteristics of the sample are reported in Table 1. Approximately half of the participants were female (56.3%), and the average age of the sample was 40.20 (SD = 14.68), with an age range between 18 and 82. The majority of participants had a degree (66.1%), were single (53.3%), and the majority were employed (63.5%) and lived in the same house with another two or three people (50.8%). In addition, most respondents reported their health status was good (39.0%) or very good (41.6%) (Table 1).

### 3.2. Impact of Social Closeness on Altruism (ISCA)

**Figure 1:**
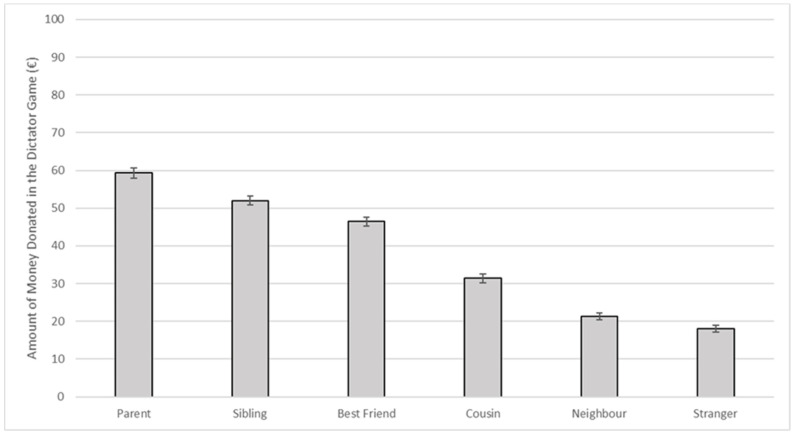
Means and standard errors of the amounts of money donated to each recipient by participants during the dictator game (*n* = 460).

The one-way repeated measures ANOVA with a Greenhouse–Geisser correction indicated that the mean donated amount of money varied significantly between the different recipients of the donation (parent, sibling, best friend, cousin, neighbour, and stranger) (*F*(3.76, 1725.11) = 424.15, *p* < 0.001, *η_p_*^2^ = 0.48). Post hoc tests using Bonferroni correction showed that all the recipients’ categories were different from each other (all *p*s < 0.001), with money donated to the parent being the highest (*M* = 59.30, *SE* = 1.37) and money donated to the stranger being the lowest (*M* = 18.11, *SE* = 0.92) (Figure 1). The ISCA index was then calculated by subtracting the amount of money donated to the stranger (the category to which participants donated the least) from the amount of money donated to the parent (the category to which participants donated the most). Higher ISCA scores indicated a higher impact of social distance on altruism (i.e., higher altruism for socially close than distant others).

### 3.3. Correlations

Pearson product-moment correlations were performed to investigate the relationship between positivity, the ISCA index, and hygiene behaviours and social distancing behaviours (Table 2). The results showed that positivity was positively associated with the ISCA index (r = 0.132, *p* = 0.004) and with COVID-19 preventive behaviours related to hygiene (r = 0.155, *p* = 0.001) but not with social distancing behaviours. Higher altruism for socially close ones (ISCA index) was associated with a higher tendency to perform COVID-19 preventive hygiene behaviours (r = 0.112, *p* = 0.016) but not with social distancing behaviours.

### 3.4. Mediation Analyses

**Figure 2:**
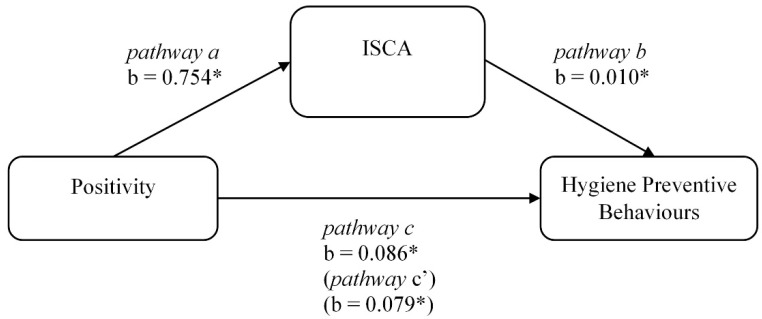
Mediation model of positivity and COVID-19 preventive behaviours related to hygiene through the impact of social closeness on altruism (*n* = 460). *Note*: b = unstandardized coefficients. * *p* < 0.05. ISCA: The impact of social closeness on altruism index.

Given the correlation results, the indirect effect of positivity in the relationship between the impact of social closeness on altruism (ISCA) was examined only for preventive hygiene behaviours. Preacher and Hayes’ [46] bootstrapping estimates of indirect effects were employed. The overall model was significant, *F*(2, 457) = 7.378, *p* < 0.001, adj. *R*^2^ = 0.031. The indirect effect of ISCA in the model was significant (unstandardized *b* = 0.007, SE = 0.005, 95% CI [0.002, 0.018]). Figure 2 displays the unstandardised regression coefficients among the model variables. Higher levels of positivity were associated with higher levels of parochialism (pathway a: unstandardised b = 0.754, 95% CI [0.235, 1.273]). Moreover, the relationship between the impact of social closeness on altruism and preventive behaviours related to hygiene was significant (pathway b: unstandardised b = 0.010, 95% CI [0.001, 0.019]). The total effect of positivity on preventive hygiene behaviours was significant (pathway c: unstandardised b = 0.086, 95% CI [0.034, 0.139]), as well as the direct effect of positivity on hygiene preventive behaviours when the impact of social closeness on altruism was included in the model (pathway c′: unstandardised b = 0.079, 95% CI [0.027, 0.132]). The result indicated that the impact of social closeness on altruism significantly and partly mediated the relation between positivity and preventive behaviours related to preventive hygiene behaviours.

## 4. Discussion

The present study explored how positivity and parochial altruism could affect COVID-19 health-protective behaviours during the Italian lockdown. The results indicated that both positivity and parochial altruism (more altruism to close vs. distant ones) were positively associated with the implementation of hygiene behaviours (such as frequent handwashing). 

Many studies indicated that positivity was related to more healthy habits and behaviours through the recourse of better adaptive coping strategies [14,22,50]. In the context of the COVID-19 pandemic, positivity was a significant predictor of death distress and happiness [25]. However, no studies showed the effect of this trait-like basic attitude on COVID-19 protective behaviours. Interestingly, we found that preventive hygiene behaviours were affected by positivity, confirming recent literature that observed different predictors for hygiene (e.g., emotional reactions) and for social distancing (e.g., self-efficacy) behaviours [51,52]. 

We did not find a significant relationship between positivity and social distancing. However, it must be noted that the study was conducted during the first total Italian lockdown when people had no liberty at all to go out for reasons other than necessity and had to respect social distancing rules strictly. Therefore, following those rules might have determined a ceiling effect and the social distancing behaviours might have been less influenced by personal characteristics such as personality traits or prosocial motivations [53,54,55].

In line with previous studies, we found that altruism was affected by social distance, with participants being more generous to close compared with distant ones [33,39,56,57]. In particular, participants donated the most generous amount of money to parents and siblings and the lowest amount of money to neighbours and strangers. In line with our findings, in the context of the COVID-19 pandemic, Van de Groep and colleagues [35] showed that adolescents who played a novel dictator game gave more money to familiar others (i.e., friends) than unfamiliar peers. Our results extend these findings to a more general sample using a more classical hypothetical dictator game.

Importantly, we found that higher parochial altruism was also associated with higher compliance with hygiene behaviours. This is in line with recent research that showed that higher altruism and empathy promote engagement in COVID-19 protective behaviours [30,31,58]. While the lack of effect on social distancing behaviours might be due, again, to a ceiling effect due to the impossibility of ignoring those rules [53,54,55], the effect found on the hygiene behaviours supports the hypothesis that the prosocial attitude towards close ones and not the population in general determines the compliance to these rules. A lower impact of social distance on altruism (more generosity towards distant ones) has been found only in extraordinary altruists such as kidney donors [34], in mothers who decide to donate umbilical blood cord to strangers [39], and in older adults who might develop more self-transcendent goals [59]. In contrast, parochial altruism seems to emerge as a consequence of warfare and between-group competition to promote individual and child fitness [60,61]. In this view, it is unsurprising that higher parochial altruism is associated with higher compliance with hygiene rules during a worldwide outbreak. Accordingly, the parasite-stress theory posits that people prefer to cooperate with in-group over out-group members since reducing contact with the out-group may be an adaptive preference for avoiding novel parasites and managing local infectious diseases [62,63]. Moreover, the in-group favouritism concentrates prosociality among family members to deal more effectively with morbidity and mortality of infectious diseases [62,64].

Finally, we found that the effect of positivity on hygiene behaviour was mediated by the increment in parochial altruism. Specifically, our mediation model showed that positivity enhanced the implementation of hygiene measures by increasing parochial altruism. This is in line with the fact that positivity equips people with abilities (such as ego-resilience, self-efficacy, and prosociality) that makes them able to face life difficulties in the best adaptive way [14]. Thus, it is not surprising that during a global outbreak, people’s levels of positivity were associated with more adaptive responses, such as an increment of parochial altruism, which, in times of warfare, is a response to promoting one’s fitness [60].

There are some limitations to this study. First, the monetary decisions used for the dictator game were hypothetical in order that we may have some ecological validity issues. However, many studies showed that participants tend to respond similarly when actual or hypothetical amounts of money are involved [38,65]. Second, we asked people to self-report the rate of altruistic and protective behaviours; therefore, results may be influenced by social desirability concerns. Third, the lockdown context limits the generalizability of the findings to other phases of the COVID-19 emergency. However, these results align with other recent studies that suggest that the predictors of protective behaviours are context-specific [54,66]. Fourth, positivity and parochial altruism explain a small portion of the variance of protective hygiene behaviours. However, we expected these results since the literature reported that many different variables significantly impacted the implementation of protective hygiene behaviours during COVID-19 pandemic lockdown (e.g., [67,68,69]). Nonetheless, our research extends the literature on the effects of prosociality on the engagement of COVID-19 protective behaviours [30,31], improving our knowledge of the determinant of compliance to protective behaviours during a specific emergency condition such as a quarantine situation.

## 5. Conclusions

The present study examined the role of two important positive attitudes, positivity and parochial altruism, on the engagement of protective behaviours during the first COVID-19 lockdown in Italy. The results indicate that both positivity and parochial altruism are related to higher hygiene preventive behaviours and that the increment of parochialism altruism mediates the effect of positivity on these behaviours. Besides the small effect size of the model, our findings expand the knowledge about the potential under-researched variables that could affect a very complex behaviour such as implementing hygiene behaviours during a pandemic and could help practitioners to take into account also these variables to enrich prevention programmes aimed at promoting the engagement of health-protective behaviours. In contrast, social distancing behaviours were not related to positivity or prosociality. Future studies are needed to investigate these same relations in other stages of the pandemic besides from the lockdown, where social distancing behaviours may have more possibility to variate.

## Figures and Tables

**Table 1:** Descriptive characteristics of the sample (*n* = 460).

Characteristic	Mean ± SD or *n* (%)
Age	40.20 ± 14.68
Sex—male	201 (43.7)
Highest education level	
High school and below	156 (33.9)
College graduates and students	205 (53.5)
Post-graduate	58 (12.6)
Marital status	
Single	245 (53.3)
Married/living with a partner	172 (37.4)
Not married	43 (9.3)
Employment—full or part-time	292 (63.5)
Number of persons in the house	2.12 ± 1.39
Self-reported health status	2.62 ± 0.81

**Table 2:** Descriptive characteristics of the sample (*n* = 460).

Variable	M	SD	1	2	3	4
1. Positivity	29.93	5.42	-			
2. Social closeness on altruism (ISCA)	41.19	30.92	0.132 **	-		
3. Hygiene behaviours	11.24	3.12	0.150 **	0.112 *	-	
4. Social distancing behaviours	14.31	2.21	0.020	0.026	0.026	-

* *p* < 0.05; ** *p* < 0.01. ISCA: The impact of social closeness on altruism index.

## Data Availability

The data that support the findings of this study are available from the corresponding author, (C.G.), upon reasonable request.
